# Novel dual-gene target and probe-based real-time PCR for the detection and differentiation of *Salmonella* Dublin

**DOI:** 10.1128/aem.01979-25

**Published:** 2026-04-16

**Authors:** Alejandra Arevalo-Mayorga, Samantha R. Locke, Mekuria Zelalem, Morgan Childs, Greg Habing

**Affiliations:** 1 Department of Veterinary Preventive Medicine, The Ohio State University 2647, Columbus, Ohio, USA; Universidad de los Andes, Bogotá, Colombia

**Keywords:** *Salmonella *Dublin, duplex qPCR, cattle, animal samples, environmental detection

## Abstract

*Salmonella* enterica serovar Dublin (*S*. Dublin) is a host-adapted cattle pathogen that causes systemic disease, chronic carriage, and environmental contamination. Early detection of subclinical infections remains challenging due to low bacterial loads and the limitations of conventional diagnostics. We developed a duplex TaqMan real-time PCR assay targeting a chromosomal locus (SeD_A1104) and a plasmid-encoded gene (*vagD*). The assay demonstrated strong linearity (*R*² > 0.99) with amplification efficiencies of 91%–102% and detection limits as low as 1.9 × 10¹ genome copies per reaction. Specificity was confirmed *in silico* and against 20 non-Dublin *Salmonella* serovars, with no cross-amplification. Intra- and inter-assay variability was consistently below 3%. Field testing of 40 samples from subclinical cattle and farm environments demonstrated detection in multiple matrices, including feces (50% positive), vaginal swabs (40%), nasal swabs (30%), and environmental boot-swabs (75%). The dual-target design provides improved confidence in detection, reduces false positives, and supports early detection and control of *S*. Dublin in cattle herds and associated environments, thereby contributing to reduced zoonotic transmission and enhancing food safety within a One Health framework.

**IMPORTANCE:**

Early detection of *Salmonella* Dublin is critical for controlling disease in cattle and reducing zoonotic risk. Existing diagnostics often fail to identify subclinical carriers or low-level environmental contamination, which sustain transmission within herds. We developed a dual-target duplex quantitative real-time PCR assay that simultaneously amplifies a chromosomal locus and a plasmid-encoded gene, improving specificity and reducing false positives caused by genetically related *Salmonella* serovars. Unlike many current tools, this assay is validated for direct use with challenging samples, including feces, nasal and vaginal swabs, and environmental boot swabs, where inhibitors and low pathogen load commonly interfere with detection. By enhancing sensitivity and reliability across diverse matrices, this method supports farm-level surveillance, rapid outbreak response, and risk-based control strategies. Broader implementation can improve herd health, limit economic losses, and reduce the risk of human exposure through food or environmental sources.

## INTRODUCTION

*Salmonella* Dublin (*S*. Dublin) is a significant concern in cattle populations due to its host-adapted nature, multidrug resistance, zoonotic potential, and challenges in detection (1–3). Unlike other *Salmonella* serovars, Dublin causes systemic infections, chronic carriage, and persistent environmental contamination through intermittent shedding (4–6). These features negatively impact animal health, increasing mortality, reducing milk production, and elevating veterinary costs, resulting in substantial economic losses (7, 8).

Furthermore, *S*. Dublin is a recognized zoonotic pathogen that causes sporadic yet severe human infections, primarily linked to the consumption of contaminated milk and beef (9, 10). Recent reports indicate an increasing incidence of human infections in North America and Europe (9, 11), emphasizing the need for improved surveillance at the interface of cattle production and public health.

The global importance of *S*. Dublin is reflected in surveillance programs in countries including Sweden and Denmark and ongoing research initiatives in the United States and Canada (12–15). However, control efforts remain hindered by the limitations of current diagnostic tools, often inadequate to detect asymptomatic carriers and environmental reservoirs, which prompt on-farm contamination and sustain infection cycles within herds (5, 6).

Current diagnostic methods, such as culture-based diagnostics, are labor-intensive and demonstrate low sensitivity for non-clinical samples (16, 17). Serological assays such as enzyme-linked immunosorbent assay can be confounded by vaccination (18, 19), which is widely practiced in U.S. dairy herds. Conventional PCR methods target markers that, although historically used for *Salmonella* detection, lack sufficient specificity for *S*. Dublin due to conserved sequences across serovars (20–22).

Quantitative real-time PCR (qPCR) improves sensitivity and speed, but most *S*. Dublin assays rely on a single target region within the *vagC* gene (23). However, homologous *vagC* sequences are present on plasmids in other Enterobacteriaceae, complicating specific detectio*n* (24). Although a commercial qPCR kit (“Salmonella 4 cows”) is available in Europe, its detection limit (~2–3 log CFU) may not suffice for identifying low pathogen loads, limiting its utility for early detection (25).

To address these limitations, we developed a duplex TaqMan qPCR assay. We hypothesize that a dual-target (chromosomal and plasmid) qPCR assay will provide high specificity and sensitivity for the detection of *Salmonella* Dublin in cattle and environmental samples. Herein, we present the analytical validation of this assay and demonstrate its application to a set of field samples collected from cattle and farm environments with histories of *S*. Dublin exposure.

## RESULTS

### Primer/probe design and *in silico* validation

**Fig 1:**
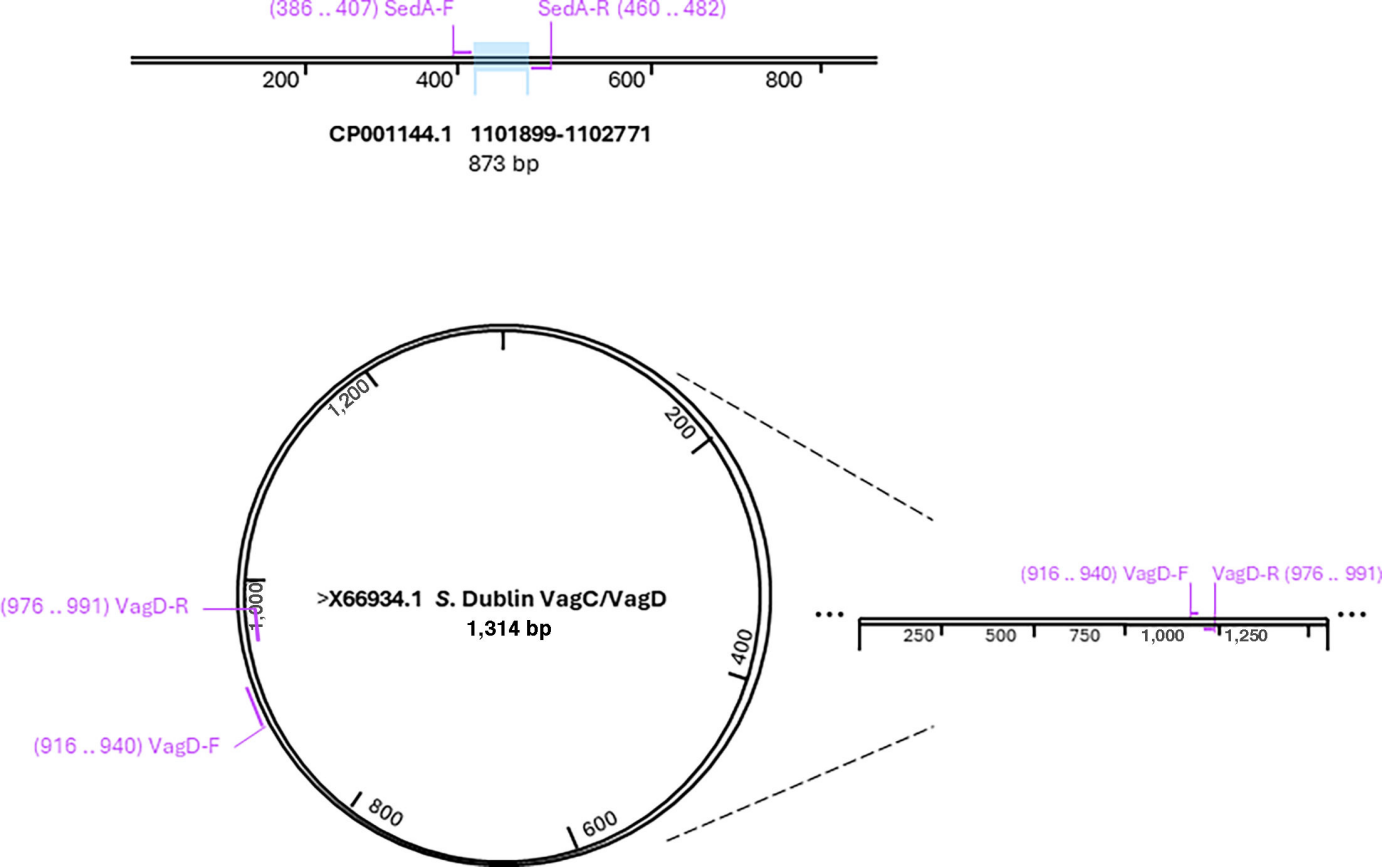
Genomic location of the targets amplified by the duplex TaqMan qPCR assay for *S*. Dublin. (Top) Schematic representation of the SeD_A1104 region on the *S*. Dublin chromosome (GenBank accession CP001144.1). Primer binding sites (Sed_A1104-F and Sed_A1104-R) are indicated, along with the predicted 97 bp amplicon. (Bottom) Circular map of the *S*. Dublin virulence plasmid (GenBank accession X66934.1), highlighting the region containing the *vagD* target. The lower panel shows a linear view of this region, with primer binding sites (vagD-F and vagD-R) and amplicon coordinates indicated. The assay targets a 76 bp fragment within the *vagC/vagD* locus.

The duplex TaqMan-based qPCR assay targets two independent genetic loci specific to *S*. Dublin: a 97 bp fragment of the chromosomal locus SeD_A1104 located within the Type VI Secretion System (SPI-6) and a 76 bp fragment of the plasmid-encoded *vagD* gene found on the virulence plasmid pSDV (see binding sites and predicted amplicon sizes in Fig. 1).

*In silico* analysis using Primer-BLAST against genomic databases confirmed that both primer sets align to their respective targets. For SeD_A1104, predicted amplicons were identified in all *S*. Dublin genomes retrieved within the first 100 Primer-BLAST hits (*n* = 39), with primers aligning in the correct orientation and distance to generate the expected 97 bp product. However, these primers also matched sequences in non-Dublin serovars, including *S*. Adjame (*n* = 7), *S*. Javiana (*n* = 8), and *S*. Senftenberg (*n* = 6).

In contrast, vagD primers demonstrated predicted binding primarily in *S*. Dublin (*n* = 78) and closely related avian-adapted serovars *S*. Gallinarum (*n* = 15) and *S*. Pullorum (*n* = 4), with limited matches in other serovars such as *S*. Enteritidis (*n* = 2) and *S*. Typhimurium (*n* = 1). However, SeD_A1104 primers did not match these latter genomes. Other serovars, including *S*. Infantis, *S*. Indiana, *S*. Milwaukee, and *S*. Schwarzengrund, exhibited mismatches at critical 3′ ends, likely impairing amplification.

The *in silico* evaluation of the duplex qPCR assay using Primer-BLAST is summarized in Table 1.

**TABLE 1:** *In silico* Primer-BLAST alignment results for SeD_A1104 and vagD primers across selected *Salmonella* serovars^*a*^

Serovar	*N*	*In silico* predicted amplification			
		SeD_A1104-F	SeD_A1104-R	vagD-F	vagD-R
Dublin	78	Expected	Expected	Expected	Expected
4,[5],12:i:-	1	Expected	Expected	Absent	Absent
Adjame	7	Expected	Expected	Absent	Absent
Agona	2	Expected	Expected	Absent	Absent
Albany	1	Expected	Expected	Absent	Absent
Bispebjerg	1	Expected	Expected	Absent	Absent
Djakarta	1	Expected	Expected	Absent	Absent
Enteritidis	7	Expected (2) Absent (5)	Expected (2) Absent (5)	Expected (2) Weak/Absent (5)	Expected (2) Weak/Absent (5)
Galinarum	15	Absent	Absent	Expected	Expected
Galinarum/Pullorum	1	Absent	Absent	Expected	Expected
Hvittingfoss	1	Expected	Expected	Absent	Absent
Indiana	3	Absent	Absent	Weak/Absent	Weak/Absent
Infantis	23	Absent	Absent	Weak/Absent	Weak/Absent
Inverness	2	Expected	Expected	Absent	Absent
Javiana	8	Expected	Expected	Absent	Absent
Karamoja	2	Expected	Expected	Absent	Absent
Kisaware	1	Expected	Expected	Absent	Absent
Milwaukee	1	Expected	Expected	Absent	Absent
Muenster	1	Expected	Expected	Absent	Absent
Newport	7	Expected	Expected	Absent	Absent
Pullorum	4	Absent	Absent	Expected	Expected
Saintpaul	2	Expected	Expected	Absent	Absent
Schwarzengrund	2	Absent	Absent	Weak/Absent	Weak/Absent
Senftenberg	8	Expected (7) Weak/Absent	Expected (7) Weak/Absent	Absent	Absent
Stanleyville	1	Expected	Expected	Absent	Absent
Typhi	1	Expected	Expected	Absent	Absent
Typhimurium	5	Expected (3) Absent (2)	Expected (3) Absent (2)	Expected (1) Weak/Absent (1) Absent (3)	Expected (1) Weak/Absent (1) Absent (3)
Uganda	1	Expected	Expected	Absent	Absent
Waycross	1	Expected	Expected	Absent	Absent

^*a*^ Results summarize predicted primer binding and amplicon generation based on the top 100 hits for each serovar. Expected = Primer aligned with correct orientation and spacing to produce the predicted amplicon. Absent = No suitable binding site detected for the primer. Weak/Absent = Mismatch detected at the 3′ end, suggesting reduced or no amplification efficiency. For serovars with multiple genome entries (*N* > 1), the number of genomes with each result type is indicated in parentheses.

### Analytical performance

The duplex qPCR assay demonstrated robust performance with both genetic targets. Standard curves generated from 10-fold serial dilutions of purified *S*. Dublin genomic DNA, ranging from approximately 3.82 × 10⁷ to 3.82 × 10^0^ genome equivalent copies per reaction, showing strong linearity, with correlation coefficients (*R*²) 0.994 for the chromosomal target (SeD_A1104) and 0.996 for the plasmid target (*vagD*).

**Fig 2:**
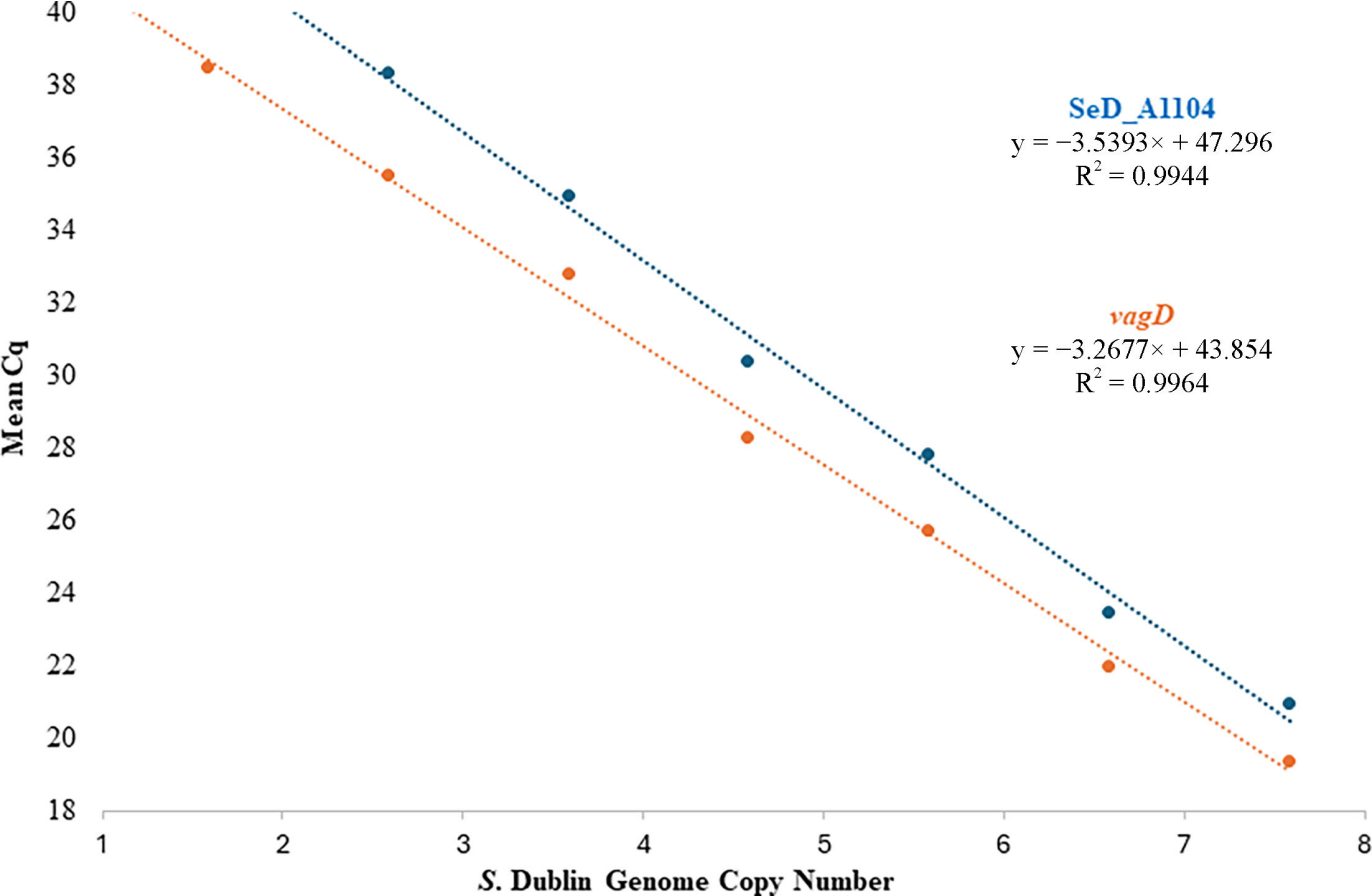
Standard curves for the SeD_A1104 and *vagD* targets of the duplex TaqMan qPCR assay for *S*. Dublin**.** Mean Cq values are plotted against the log₁₀-transformed genome equivalent copies per reaction. Linear regression, *R*² values, and amplification efficiencies are shown for each target.

Amplification efficiencies were calculated at 91.67% for SeD_A1104 and 102.3% for *vagD*, with corresponding slopes of −3.54 and −3.27, indicating efficient PCR amplification across the dilution range (Fig. 2).

The limit of detection (LOD), defined as the lowest concentration consistently detected in all replicates, was 191 genome equivalent copies per reaction for SeD_A1104 (mean Cq = 38.32) and 19 genome equivalent copies per reaction for *vagD* (mean Cq = 39.17). All replicates tested positive at these concentrations, demonstrating high sensitivity.

The limit of quantification (LOQ) was defined as the lowest concentration showing acceptable precision and consistent Cq values across replicates. For both targets, the LOQ matched with the LOD, reflecting reliable quantification at these low concentrations. The assay demonstrated reliable quantification over six orders of magnitude for SeD_A1104 and seven for *vagD*, with consistent amplification and linear response across the tested range. Amplification of the internal positive control (IPC) was consistent in all reactions (mean Cq = 12.77 ± .32), indicating the absence of PCR inhibition during assay validation.

Precision assessments showed low variability in both intra-assay and inter-assay testing. Intra-assay precision was assessed using purified *S.* Dublin genomic DNA at 5 ng per reaction (approximately 9.5 × 10 ^5^ genome equivalent copies), with three technical replicates per target in a single qPCR run (Table 2). The chromosomal target (SeD_A1104) showed a mean Cq of 24.50 ± 0.27 (standard deviation [SD]) with a coefficient of variation (CV) ranging from 0.70% to 1.99%. For the plasmid target (*VagD*), mean Cq was 21.94 ± 0.33 (SD), with CV between 0.68% and 1.86%.

**TABLE 2:** Intra-assay variation for SeD_A1104 and *VagD* targets^*a*^

Assay	SeD_A1104				vagD			
	Cq	Mean Cq	SD (Cq)	CV (%)	Cq	Mean Cq	SD (Cq)	CV (%)
1	26.65	26.76	0.19	0.70	24.03	24.24	0.52	2.13
	26.65				23.86			
	26.98				24.83			
2	24.06	24.04	0.18	0.77	21.07	21.60	0.15	0.68
	23.84				20.84			
	24.21				21.41			
3	24.96	24.47	0.49	1.99	20.88	20.60	0.28	1.34
	24.47				20.60			
	23.98				20.32			
4	24.08	24.21	0.22	0.92	22.61	22.14	0.41	1.86
	24.47				21.89			
	24.09				21.92			
5	23.01	23.05	0.28	1.19	21.07	21.11	0.29	1.36
	22.79				20.84			
	23.34				21.41			

^*a*^ Mean Cq, standard deviation (SD), and coefficient of variation (CV%) were calculated for each target across five independent runs using *S*. Dublin pure DNA.

Inter-assay reproducibility, evaluated across five independent qPCR runs on separate days by different individuals, yielded an overall mean Cq values of 24.50 ± 0.27 (CV 1.12%) for SeD_A1104 and 21.94 ± 0.33 (CV 1.47%) for *VagD*. All CV values remained below 3%, indicating high precision and consistent assay performance under routine laboratory conditions.

#### Analytical specificity and inclusivity

**Fig 3:**
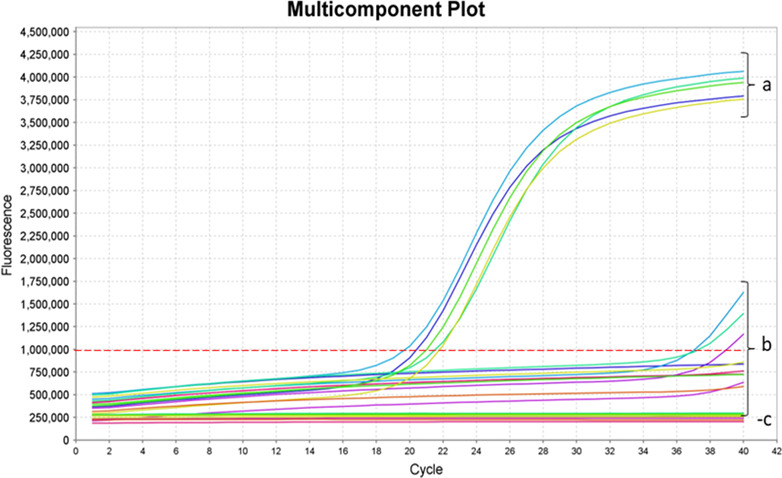
Fluorescence amplification curves generated by the TaqMan qPCR assay. The dashed red line indicates the fluorescence threshold. (**a**) Amplification curves for *S.* Dublin isolates. (**b**) Reactions with non-Dublin *Salmonella* serovars. (**c**) No-template controls (NTCs). Blue, green, and pink solid lines represent amplification curves for the *vagD* target in *S*. Agona, *S*. London, and *S*. Enteritidis, respectively.

Assay specificity was evaluated using genomic DNA from 10 confirmed *S*. Dublin isolates, representative of the limited genomic heterogeneity observed within this serovar based on whole-genome sequencing analysis (Fig. S1), and 20 non-target *Salmonella* serovars (Table 3, Fig. 3). All *S*. Dublin isolates consistently produced amplification with both targets. The plasmid-encoded (*vagD*) showed Cq values ranging from 20.8 to 24.9, while the chromosomal target (SeD_A1104) had Cq values ranging from 22.0 to 24.8. These isolates originated from diverse cattle sources, including bovine lung, intestinal tissue, lymph nodes, and environmental samples from slaughterhouses and veal operations.

**TABLE 3:** Bacterial isolates for inclusivity and exclusivity testing of the duplex qPCR assay^*a*^

*Salmonella* serovar	Strain ID and accession no.	Source/origin	Target	Mean Cq	Result
Dublin	SD-5354 SAMN46721045	Lung (2012) – Clinically ill bovine	SeD_A1104	25.21	Positive
			*vagD*	22.47	
Dublin	SD-36421 SAMN08744620	Lung (2013) – Clinically ill bovine	SeD_A1104	23.36	Positive
			*vagD*	20.99	
Dublin	SD-38950 SAMN08744638	Lung (2014) – Clinically ill bovine	SeD_A1104	25.22	Positive
			*vagD*	21.85	
Dublin	SD-143 SAMN46720611	Intestine (2015) – Clinically ill bovine	SeD_A1104	23.73	Positive
			*vagD*	21.04	
Dublin	ENV-60 SAMN12715320	Environment (2019) – Slaughterhouse floor	SeD_A1104	21.24	Positive
			*vagD*	20.24	
Dublin	ENV-223 SAMN15734986	Environment (2019) – Veal barn, boot swab	SeD_A1104	23.56	Positive
			*VagD*	20.33	
Dublin	SF-435M1 SAMN35723248	Lymph node (2022) – Special-fed veal	SeD_A1104	22.19	Positive
			*vagD*	20.12	
Dublin	SF-552SB1 SAMN35723253	Lymph node (2022) – Special-fed veal	SeD_A1104	23.06	Positive
			*vagD*	20.53	
Dublin	DB-261SB1 SAMN47319713	Lymph node (2023) – Dairy beef steers	SeD_A1104	22.90	Positive
			*vagD*	20.26	
Dublin	DB-400RV1 SAMN46114380	Lymph node (2023) – Dairy beef steers	SeD_A1104	21.02	Positive
			*vagD*	20.41	
Agbeni	DB-11M1 SAMN47225999	Lymph node (2022) – Dairy beef steers	SeD_A1104	Undetermined	Negative
			*vagD*		
Agona	ENV-04B SAMN12715331	Environment (2018) – Livestock trailer floor	SeD_A1104	37.6	Negative
			*vagD*	37.5	
Blockley	SF-659RV1 SAMN46114389	Lymph node (2022) – Special-fed veal	SeD_A1104	Undetermined	Negative
			*vagD*		
Barranquilla	DB-198M1 SAMN47226011	Lymph node (2022) – Dairy beef steers	SeD_A1104	Undetermined	Negative
			*vagD*		
Cerro	MAT-54RV1 SAMN47226240	Environment (2023) – Dairy farm, maternity pen	SeD_A1104	Undetermined	Negative
			*vagD*		
Schwarzengrund	BV-42 AMSL-OSU	Lymph node (2021) – Bob veal	SeD_A1104	Undetermined	Negative
			*VagD*		
Enteritidis	SENT-30542 SAMN10581948	Feces (2015) – Clinically ill cattle, ODA-ADDL	SeD_A1104	Undetermined	Negative
			*vagD*	38.7	
Give	ENV-249A SAMN17964394	Environment (2019) – Livestock trailer floor, CDC surveillance	SeD_A1104	Undetermined	Negative
			*vagD*		
Infantis	SF-648M1 SAMN47226038	Lymph node (2022) – Special-fed veal	SeD_A1104	Undetermined	Negative
			*vagD*		
Kiambu	DB-134M2 SAMN47319711	Lymph node (2022) – Dairy beef steers	SeD_A1104	Undetermined	Negative
			*vagD*		
London	DB-189S2 SAMN51795269	Lymph node (2022) – Dairy beef steers	SeD_A1104	38.8	Negative
			*vagD*	37.7	
Montevideo	DB-365M1 SAMN47226021	Lymph node (2023) – Dairy beef steers	SeD_A1104	Undetermined	Negative
			*vagD*		
Muenster	ENV-149 SAMN17964381	Environment (2019) – Slaughterhouse floor, CDC surveillance	SeD_A1104	Undetermined	Negative
			*vagD*		
Newport	SN-12065 SAMN46721677	Placenta (2013) – Clinically ill cattle, ODA-ADDL	SeD_A1104	Undetermined	Negative
			*vagD*		
Oranienburg	DB-130RV1 SAMN47226007	Lymph node (2022) – Dairy beef steers	SeD_A1104	Undetermined	Negative
			*vagD*		
Panama	LSM-191 SAMN15734989	Environment (2019) – Livestock market alleyway	SeD_A1104	Undetermined	Negative
			*vagD*		
Ruiru	DB-212RV1 SAMN47226012	Lymph node (2023) – Dairy beef steers	SeD_A1104	Undetermined	Negative
			*vagD*		
Rubislaw	SF-177RV1 SAMN46114382	Lymph node (2022) – Special-fed Veal	SeD_A1104	Undetermined	Negative
			*vagD*		
Typhimurium	ST-36 SAMN46721679	Colon (2018) – Clinically ill cattle	SeD_A1104	Undetermined	Negative
			*VagD*		
Uganda	ENV-161 AMSL-OSU	Environmental (2019) – Livestock trailer floor, CDC surveillance	SeD_A1104	Undetermined	Negative
			*vagD*		

^*a*^ The panel includes whole-genome sequencing (WGS)-confirmed *S.* Dublin strains (inclusivity) and other *Salmonella* serovars (exclusivity). For each isolate, Cq values for each target (SeD_A1104 and *vagD*) are reported, along with the overall qPCR interpretation (Cq < 35 = positive; >35 or undetermined = negative). WGS, whole-genome sequencing; Cq, quantification cycle; SeD_A1104, chromosomal locus target; *vagD*, plasmid-encoded gene target. Strain ID refers to the laboratory identifier. Accession number refers to the NCBI database identifier. AMSL-OSU refers to the IDI Applied Microbiology Services Laboratory (AMSL) at The Ohio State University.

No true amplification was detected in any of the non-Dublin strains. Weak and delayed signals were observed in *S*. Agona for both targets, with late mean Cq values (37.6 for *vagD* and 37.5 for SeD_A1104), but these lacked the characteristic sigmoidal curve of true amplification. Likewise, *S*. London and *S*. Enteritidis generated weak signals for the *vagD* target only (mean Cq = 37.7), also considered non-specific due to the absence of typical amplification curves and the Cq values exceeding the positivity threshold (Cq ≤ 35).

Amplicon identity was further confirmed by Sanger sequencing of representative PCR products from *S*. Dublin isolates, showing 100% identity to the reference *vagD* and SeD_A1104 regions.

### Application to field samples

The duplex qPCR assay was applied to 40 field samples of unknown status, including nasal swabs, vaginal swabs, and feces from asymptomatic dairy cows with a known history of *S*. Dublin exposure, as well as environmental boot swabs collected from dairy-beef and veal farms. Among animal samples, feces showed the highest positivity rate (5/10, 50%), followed by vaginal swabs (4/10, 40%) and nasal swabs (3/10, 30%) (Table 4). Overall, *S*. Dublin was detected in at least one sample type from 6 out of 10 cows (60%). Two cows were positive across all three sample types.

**TABLE 4:** Detection of *S*. Dublin by duplex qPCR across different sample types collected from cattle and cattle environment^*a*^

Sample type	% Positive	Mean Ct (± SD) SeD_A1104	Mean Ct (± SD) *vagD*	IPC Ct (± SD) *vagD*
Nasal swabs (*n* = 10)	30% (3/10)	28.673 ± 0.07	25.098 ± 0.15	20.11 ± 0.76
Vaginal swabs (*n* = 10)	40% (4/10)	34.155 ± 0.97	29.434 ± 3.31	18.16 ± 1.83
Feces (*n* = 10)	50% (5/10)	30.971 ± 3.5	29.133 ± 4.02	16.086 ± 1.01
Environmental boot swabs (*n* = 8)	75% (6/8)	30.02 ± 1.92	29.22 ± 2.70	18.132 ± 3.70

^*a*^ % Positive = proportion of positive samples per sample type. Mean Ct (± SD) = average cycle threshold (Ct) values with standard deviation (SD) for each gene target (SeD_A1104 and *vagD*) among positive samples only.

Environmental samples consisted exclusively of boot swabs collected along the perimeter of calf hutches housing pre-weaned calves and within barns holding post-weaned calves. Six of these samples showed positive amplification for both the SeD_A1104 and *vagD* targets (Cq < 35), indicating detectable environmental contamination (Table 4). Two boot swabs exhibited PCR inhibition, evidenced by the failure of the internal amplification control (16S rRNA gene), and were therefore excluded from analysis. Considering only the eight analyzable samples, the positivity rate was 75% (6/8). The occurrence of inhibition in a subset of boot swab samples is consistent with the presence of environmental inhibitors (e.g., organic material and disinfectants) and highlights the importance of including internal controls to ensure reliable interpretation.

## DISCUSSION

This study presents the development and analytical validation of a duplex TaqMan qPCR assay, alongside preliminary field evaluation, for specific detection and quantification of *S*. Dublin in cattle and associated environmental samples. The assay’s dual-target strategy ensures positive identification requires concurrent signals from two independent genomic loci, enhancing specificity and reducing false positives. A 35-cycle cut-off was applied following commonly used qPCR guidelines (26), which recommend excluding late-cycle amplification to reduce the likelihood of non-specific or spurious signals (26).

Given the close genetic relationships among *Salmonella* serovars, designing highly specific qPCR primers remains challenging, particularly due to the constraints on amplicon size (50–150 bp) necessary for efficient amplification in complex sample matrices (27). Incorporating multiple genetic targets offers a robust approach to mitigate false positives arising from target overlap or sequence variability. Our *in silico* analyses confirmed consistent detection of both targets across all *S*. Dublin genomes examined, although SeD_A1104 exhibited limited exclusivity with predicted amplification in several non-target serovars. However, empirical testing with relevant *Salmonella* strains, including Enteritidis and Typhimurium, resulted in no amplification or non-specific signals, aligning with previous reports (28, 29). This underscores that theoretical primer binding does not always translate into meaningful assay amplification under lab conditions.

The inclusion of SeD_A1104 is warranted due to its repeated identification as an *S*. Dublin-associated marker (28–30), complemented by the plasmid target *vagD*, which exhibits strong exclusivity. Although vagD primers show homology to avian-adapted serovars Gallinarum and Pullorum, eradication of these pathogens from US commercial poultry and their rare association with cattle minimize the risk of diagnostic interference (31).

Analytical validation demonstrated high assay efficiency (91.7% to 102.3%) and excellent linearity (*R*² > 0.99) across a broad dynamic range. When viewed in the context of published detection limits for assays targeting *vagC* or for commercial assays developed in Europe (23, 25), the analytical sensitivity achieved here provides a useful benchmark for interpreting assay performance, acknowledging that no direct head-to-head comparisons were performed (23, 25). The higher analytical sensitivity observed for the plasmid-encoded *vagD* target may partially reflect increased template abundance relative to chromosomal loci, although, to our knowledge, the precise copy number of the *S*. Dublin virulence plasmid has not been determined. Overall, enhanced sensitivity facilitates detection of carriers, which play critical roles in within-herd and environmental transmission (6, 32, 33).

Preliminary field testing of 30 samples collected from healthy cattle demonstrated that the duplex assay performs reliably across multiple animal-derived matrices, including feces, vaginal and nasal swabs. Although the sample size was limited and not intended for statistical inference, these results provide proof of concept for detecting *S*. Dublin in sample types where low pathogen loads can pose detection challenges. Larger studies will be valuable for formally assessing diagnostic performance, but the current findings support the suitability of the assay for animal-level surveillance in cattle production systems.

Environmental samples may contain organic debris, which introduces inhibitors such as humic and fulvic acids (34), as well as disinfectant residues that can interfere with nucleic acid detection by affecting nucleic acid integrity or recovery (35). In this study, 2 out of 10 boot swab samples showed inhibition, evidenced by failure of the internal control, while the remaining samples amplified successfully. Occasional inhibition is therefore expected in such matrices and underscores the importance of including an internal control throughout the process to monitor inhibition and amplification integrity. However, the inhibition occurred only in a subset of samples, indicating a heterogeneous distribution of inhibitors rather than a limitation of the duplex assay itself. While *S*. Dublin currently causes relatively infrequent zoonotic infections, emerging evidence indicates a rising incidence in humans (9, 11). By enabling early detection of subclinical carriers and on-farm environmental contamination, this assay contributes to the interruption of zoonotic transmission chains. Thus, its application supports infectious disease control beyond agriculture, improving public health outcomes.

Implementing this assay in diagnostic labs may provide efficiency and cost benefits, supporting rapid, high-throughput testing and complex sample pooling, making it a valuable addition to comprehensive surveillance strategies. Future work should focus on refining genetic targets to resolve existing specificity limitations and expanding field validations across diverse production systems to solidify its diagnostic utility and epidemiological impact.

In conclusion, this duplex qPCR assay offers a sensitive, specific, and practical diagnostic option for detecting *S*. Dublin in clinical and environmental samples, leveraging two independent targets to improve confidence and reduce false positives. This supports better early identification of infected animals (individual level) and contaminated environments (farm-level), advancing biosecurity, outbreak control, and public health protection efforts for S. Dublin in cattle production.

## MATERIALS AND METHODS

The assay development followed the Minimum Information for Publication of Quantitative Real-Time PCR Experiments (MIQE) guidelines (36).

### Bacterial strains

Thirty isolates (*n* = 10 *S*. Dublin; *n* = 20 non-Dublin serovars; Table 3) were selected for inclusivity and exclusivity testing. Isolates were obtained from cattle or cattle environments in prior research conducted by the One Herd Lab (OSU) or were provided by collaborating Veterinary Diagnostic Laboratories. Each isolate was revived on Xylose-Lysine-Tergitol 4 agar (Oxoid, Hampshire, UK), with colonies subcultured onto Tryptic Soy Agar (Oxoid) and incubated at 37°C for 18–24 h to confirm purity. Strain identity was verified by whole-genome sequencing, serotyping, or reference laboratory protocols, as appropriate.

### DNA isolation

Genomic DNA was extracted using the ZymoBIOMICS DNA Miniprep Kit (Zymo Research, Irvine, CA, USA), following the manufacturer’s protocol without modification. DNA purity was assessed via spectrophotometry using a NanoDrop OneC spectrophotometer (Thermo Fisher Scientific, Waltham, MA, USA), and concentration was determined using a Qubit 4 Fluorometer (Thermo Fisher Scientific, Waltham, MA, USA). Only DNA with an OD 260/280 ratio between 1.8 and 2.0 was subsequently standardized to the concentrations required for downstream testing. Extracted DNA was stored at −20°C prior to qPCR analysis.

### qPCR assay design, conditions, and target verification

The duplex TaqMan real-time PCR assay was developed to specifically detect *S*. Dublin by targeting two genetic loci: a 97 bp fragment of the chromosomal locus SeD_A1104 (coordinates 1,101,899–1,102,771; GenBank accession CP_001144.1) within the Type VI Secretion System encoded on *Salmonella* Pathogenicity Island 6 (SPI-6) and a 76 bp fragment of the plasmid-encoded *vagD* gene, located between nucleotides 916 and 991 of the *vagC/vagD* locus on the serovar Dublin virulence plasmid pSDV (GenBank accession X66934.1).

*In silico* analyses of primer and probe specificity across *Salmonella* genomes were performed using Primer-BLAST against the NCBI database. Binding sites and amplicon sizes were verified with SnapGene Viewer version 7.0.2. To assess conservation of the diagnostic targets, we also reviewed the first 100 Primer-BLAST hits for each primer set (187 unique genome assemblies after removal of duplicates), and both loci were consistently detected across all *S*. Dublin genomes within this data set. Details of this *in silico* validation, including predicted sensitivity and cross-reactivity, are reported in the Results section.

Primers and hydrolysis probes were custom-designed (Thermo Fisher Scientific; Table 5). The SeD_A1104 probe was labeled with FAM and the vagD probe with ABY, both of which were quenched with NFQ. An internal positive control (IPC) assay targeting the bacterial 16S rRNA gene was included using a previously published primer/probe set (Ba0493079_s1) labeled with VIC-PL to monitor PCR efficiency and potential inhibition.

**TABLE 5:** Primers, hydrolysis probes, target genes, and expected amplicon sizes for the detection of *S*. Dublin by TaqMan qPCR

Primers F - R^*a*^	Target	Product size
F (5′-CCTCTGATGCCGTGATCTCTCT-3′ R (5′-GTGCTGAACACATCCAGACAATC-3′ Probe: FAM-CTCCGCAAAATTCCAC-NFQ	SeDA_1104	97 bp
F (5′-CTCGATACCTGTATCTGTTCGTTCA-3′ R (5′-GCACCGCCTGCTCAAG-3′ Probe: ABY-CTTCAGCACCGCTTCC-NFQ	*vagD*	76 bp

^*a*^ F, forward primer; R, reverse primer.

Each qPCR run included a positive control (purified S. Dublin 5354) and a no-template control (NTC). A sample was classified as positive for *S*. Dublin if the following conditions were met for both targets: (i) the amplification curve exhibited a typical sigmoidal (S-shaped) profile; (ii) the quantification cycle (Cq) value for each target was ≤35, and (iii) the internal positive control (IPC; 16S rRNA) amplified with expected efficiency (Cq in the range observed for uninhibited reactions). A cycle threshold (Cq) value of ≤35 was used to define a positive result, consistent with standard qPCR practice for detecting low-level targets with acceptable specificity and sensitivity (26)

Results were interpreted based on Cq values and expressed as estimated genome-equivalent copies per reaction, calculated from a standard curve. Amplicon identity was verified by Sanger sequencing of two representative samples per target from reference isolates.

### Limit of detection (LOD), limit of quantification (LOQ), and dynamic range

The limit of detection (LOD) and limit of quantification (LOQ) for the duplex qPCR assay were determined using genomic DNA extracted from *S*. Dublin 5354 (Ohio Department of Agriculture - Animal Disease Diagnostic Laboratory). Standard curves were constructed using sevenfold serial dilutions ranging from 100 ng/reaction to 0.00001 ng/reaction. Genome equivalent copies per reaction were calculated based on a *Salmonella* Dublin genome size of 4.87 Mb.

The limit of detection (LOD) was defined as the lowest DNA concentration at which ≥95% of replicates yielded a positive amplification with a quantification cycle (Cq) value < 35. The limit of quantification (LOQ) was established as the lowest concentration with a coefficient of variation (CV%) ≤35% across the triplicates, representing reliable quantification. Amplification efficiency (E) and correlation coefficient (*R*²) were calculated from the slope of the standard curve generated across the dilution range, encompassing approximately 6 to 7 orders of magnitude.

### Intra- and inter-assay variation

The precision of the duplex qPCR assay was assessed through intra-assay (repeatability) and inter-assay (reproducibility) variation analyses. For intra-assay testing, five *S*. Dublin isolates (5354, ENV-60B, SD-143, SF-435 M1, and DB-400) and five non-Dublin *Salmonella* serovars (Enteritidis 8612, Infantis SF-648M1, Panama LSM191, Typhimurium ST-36, and London SF-448) were run in technical triplicates within a single assay to evaluate within-run consistency.

Inter-assay variation was determined by testing the same isolates across four independent qPCR runs performed on different days, maintaining identical reaction conditions and protocols to assess between-run reproducibility. Cq values were recorded for all replicates, with mean, standard deviation (SD), and coefficient of variation (CV%) calculated to quantify variability. Acceptable precision was set at CV% ≤ 5%–10%, consistent with established quantitative PCR performance standards.

### Analytical specificity and inclusivity

Analytical specificity (exclusivity testing) of the duplex qPCR assay was assessed using a panel of 20 non-*Salmonella* Dublin serovars, including epidemiologically relevant strains such as *S*. Enteritidis, *S*. Typhimurium, and *S*. Infantis, sourced from diverse clinical and environmental contexts. For inclusivity evaluation, the assay was tested against the reference strain SD-5354 and 10 additional *S*. Dublin isolates obtained between 2012 and 2023 from clinical, non-clinical, and environmental samples (see Table 3). The presence or absence of amplification, along with Cq value, was recorded to confirm assay specificity and inclusivity according to predefined criteria. DNA was extracted and standardized to uniform concentrations (5 ng/µL) before triplicate qPCR testing under identical assay conditions.

### Sample testing

To evaluate the applicability of the duplex qPCR, previously extracted DNA from 30 biological samples, including 10 nasal swabs, 10 vaginal swabs, and 10 fecal samples, collected from asymptomatic dairy cows on a commercial dairy farm with a history of endemic *S*. Dublin infection, were tested. No animals were sampled for the present study; all DNA extracts originated from an unrelated, Institutional Animal Care and Use Committee-approved project.

Additionally, DNA extracted from 10 environmental boot swabs collected in a separate study on veal and dairy-beef farms was analyzed; these samples had been previously screened using an in-house, serovar-specific PCR. Only DNA samples with adequate quality (OD 260/280 of 1.8–2.0 and concentration ≥1 ng/µL) were included. The duplex qPCR assay was performed under the standardized conditions described above, and samples were interpreted as positive when both targets amplified with a Cq <35 and a proper sigmoidal curve.

Detailed qPCR results from these samples are provided in Table S1.

## SUPPLEMENTAL MATERIAL

Table S1 and Fig. S1 aem.01979-25-s0001.docx qPCR individual testing results and phylogenetic tree.

ASM does not own the copyrights to Supplemental Material that may be linked to, or accessed through, an article. The authors have granted ASM a non-exclusive, world-wide license to publish the Supplemental Material files. Please contact the corresponding author directly for reuse.
