# Supplementary material for: Novel dual-gene target and probe-based real-time PCR for the detection and differentiation of Salmonella Dublin
Source: Appl Environ Microbiol. 2026 Apr 16;92(5):e01979-25. doi: 10.1128/aem.01979-25 (PMC13188875; doi:10.1128/aem.01979-25)
Supplement: Table S1 and Fig. S1 — qPCR individual testing results and phylogenetic tree. [file aem.01979-25-s0001.docx]

**Supplementary Table 1.**

**Table 1-A.** Individual (animal-level) results of duplex qPCR assay and ELISA from nasal, vaginal, and fecal samples collected from 10 dairy cows. Duplex qPCR targeting the chromosomal marker SeD_A1104 and the plasmid marker *vagD* was applied to nasal swabs, vaginal swabs, and feces. Results are presented as mean quantification cycle (Cq) values across triplicates and interpreted for *S*. Dublin detection. Neg = negative result, Pos = positive result, ND = no amplification detected.

|  |  | **Nasal swabs** | | **Vaginal swabs** | | **Feces** | |
| --- | --- | --- | --- | --- | --- | --- | --- |
| Animal | Marker | Mean Cq | *S*. Dublin | Mean Cq | *S*. Dublin | Mean Cq | *S*. Dublin |
|  |  |  |  |  |  |  |  |
| Cow #1 | SeD_A1104 | 35.79 | Neg | ND | Neg | ND | Neg |
|  | VagD | 33.23 |  | ND |  | ND |  |
| Cow #2 | SeD_A1104 | 29.67 | Pos | 34.43 | Pos | 36.78 | Neg |
|  | VagD | 25.1 |  | 32.12 |  | 31.83 |  |
| Cow #3 | SeD_A1104 | 35.48 | Neg | 32.04 | Neg | 27.42 | Pos |
|  | VagD | 35.22 |  | 36.09 |  | 23.86 |  |
| Cow #4 | SeD_A1104 | 35.13 | Neg | 33.2 | Pos | 30.63 | Pos |
|  | VagD | 38.91 |  | 25.33 |  | 27.66 |  |
| Cow #5 | SeD_A1104 | ND | Neg | 36.3 | Neg | 34.75 | Pos |
|  | VagD | 35.64 |  | 35.92 |  | 33.69 |  |
| Cow #6 | SeD_A1104 | 32.46 | Pos | 29.65 | Pos | 31.6 | Pos |
|  | VagD | 30.17 |  | 30.17 |  | 32.33 |  |
| Cow #7 | SeD_A1104 | ND | Neg | 36.67 | Neg | 35.55 | Neg |
|  | VagD | 34.99 |  | 36.08 |  | 33.78 |  |
| Cow #8 | SeD_A1104 | 38.84 | Neg | 36.08 | Neg | 36.9 | Neg |
|  | VagD | ND |  | 35.9 |  | 33.25 |  |
| Cow #9 | SeD_A1104 | 28.88 | Pos | 31.89 | Pos | 30.78 | Pos |
|  | VagD | 28.23 |  | 31.94 |  | 30.1 |  |
| Cow #10 | SeD_A1104 | ND | Neg | 36.65 | Neg | 37.03 | Neg |
|  | VagD | 36.84 |  | 35.86 |  | 34.27 |  |

**Table 1-B**: Individual results of duplex qPCR assay from environmental boot-swabs collected on dairy-beef or veal farms. Conventional PCR was used to screen samples for the presence of *Salmonella* Dublin. Results for the duplex qPCR targeting the chromosomal marker SeD_A1104 and the plasmid marker *vagD* are presented as mean quantification cycle (Cq) across triplicates. Neg = negative result, Pos = positive result, Inh = inhibition (internal positive control indicated inhibition of DNA amplification).

| **Enviro sample** | **m-PCR for *S*. Dublin** | **Marker** | **Mean Cq** | **qPCR for *S*. Dublin** |
| --- | --- | --- | --- | --- |
|  |  |  |  |  |
| Dairy beef farm #1 | Pos | SeD_A1104 | 32.51 | Neg |
|  |  | VagD | 35.05 |  |
| Dairy beef farm #2 | Pos | SeD_A1104 | 31.16 | Pos |
|  |  | VagD | 28.05 |  |
| Dairy beef farm #3 | Pos | SeD_A1104 | 28.18 | Pos |
|  |  | VagD | 25.99 |  |
| Dairy beef farm #4 | Pos | SeD_A1104 | 32.37 | Pos |
|  |  | VagD | 32.54 |  |
| Dairy beef farm #5 | Pos | SeD_A1104 | 32.37 | Inh |
|  |  | VagD | 32.54 |  |
| Dairy beef farm #6 | Neg | SeD_A1104 | 31.79 | Inh |
|  |  | VagD | 30.09 |  |
| Dairy beef farm #7 | Neg | SeD_A1104 | 31.66 | Pos |
|  |  | VagD | 31.38 |  |
| Dairy beef farm #8 | Neg | SeD_A1104 | 35.22 | Neg |
|  |  | VagD | 36.93 |  |
| Veal farm #1 | Neg | SeD_A1104 | 28.56 | Pos |
|  |  | VagD | 26.63 |  |
| Veal farm #2 | Neg | SeD_A1104 | 28.18 | Pos |
|  |  | VagD | 30.73 |  |

**Supplementary Figure 1.**

Whole-genome–based phylogenetic tree of Salmonella Dublin isolates used in this study, showing limited genomic heterogeneity. Branch lengths represent nucleotide substitutions per site across the core genome. ATCC39184 was included as the reference genome.
